# Differences in the Efficiency Between the Grab and Track Starts for Both Genders in Greek Young Swimmers

**DOI:** 10.2478/v10078-012-0022-8

**Published:** 2012-05-30

**Authors:** Vassilios Thanopoulos, Georgia Rozi, Tomislav Okičić, Milivoj Dopsaj, Bojan Jorgić, Dejan Madić, Saša Veličković, Zoran Milanović, Fani Spanou, Emilios Batis

**Affiliations:** 1 Department of Aquatic Sports, Faculty of Physical Education and Sports, National and Kapodistrian,University of Athens, Athens, Greece.; 2 Faculty of Sports and Physical Education, University of Nis, Nis, Serbia.; 3 Faculty of Sport and Physical Education, University of Belgrade, Belgrade, Serbia.; 4 Swimming Club,, Paleo Faliro, Athens.

**Keywords:** swimming, start technique, kinematic analysis, flight parameters

## Abstract

The aim of this study was to determine the differences in the kinematic parameters between the grab and track starts and the differences in these two starts between genders. A total of 27 swimmers at the competitive level participated in the study, 13 boys (mean ± SD: age 15.8 ± 0.8 years, body mass 67.7 ± 7.7 kg and body height 178.6 ± 5.7 cm) and 14 girls (mean ± SD: age 16 ± 0.8 years, body mass 59.2 ± 6.6 kg and body height 166.2 ± 6.7 cm). Each swimmer performed three attempts for both start techniques. The best attempt of the grab start and the track start was taken for further analysis. The following kinematic parameters were analysed: flight distance, flight time, flight velocity, entry angle and reaction time. The males had greater numeric values for the results in all kinematic parameters for the grab start compared with the track start, except for flight velocity and entry angle (flight time 0.42 vs. 0.41 s, flight distance 3.21 vs. 3.14 m, flight velocity 7.76 vs. 7.83 m/s, entry angle 44.22 vs. 43.85 degrees and reaction time 0.86 vs. 0.81 s). The females also had greater numeric values for the results in all kinematic parameters for the grab start compared with the track start, except for flight time (flight time 0.38 vs. 0.38 s, flight distance 2.82 vs. 2.73 m, flight velocity 7.47 vs. 7.31 m/s, entry angle 45.18 vs. 44.79 degrees and reaction time 0.88 vs. 0.82 s). These results indicate that the males had significantly better results for flight time and flight distance compared with the females for the grab start (flight time 0.42 vs. 0.38 s, flight distance 3.21 vs. 2.82 m). In the case of the track start, the males had significantly better results for flight distance (3.14 vs. 2.73 m). Exploring the characteristics of the two starts did not lead to any significant kinematic differences. Therefore, a conclusion that demonstrates the superiority of one of the techniques cannot be reached. The coach, together with each swimmer individually, should devote some time to decide after some tests what type of start is better for the body type and general qualifications of the swimmer.

## Introduction

In competitive swimming, a swimmer’s time is equal to the time spent starting, stroking and turning (Hay, 1993). As world records continue to fall in many swimming events, each element of the race takes on critical importance in determining the outcome. Swimming performance is determined by many factors. The ability to perform the different technical tasks assumes critical importance. In swimming, we should distinguish at least three technical domains: starting, stroking and turning (Hay, 1986). Despite the general recognition of the importance of starts and turns, much more research has been devoted to the study of the stroking techniques (Vilas-Boas et al., 2002). Although the time a swimmer spends starting is less than in the free swimming and turning phases, an effective start is important for success (Honda et al., 2010).

The start takes up an increasing proportion of the total duration of the competition, especially if the distance is shorter (Nikodelis and Kollias, 2003). In the 50 and 100 m swimming races, performance has been strongly linked to start performance (Mason and Cossor, 2000). Hay (1986) estimated that the start accounted for 11% of the total race time for the 50 m freestyle. Start performance is defined as the time observed between the start signal and the moment when the swimmer’s head reaches 10 m (Arellano et al., 1996) or 15 m (Issurin and Verbitsky, 2002; Mason and Cossor, 2000). One of the first studies producing many different types of data during the swimming start was developed by Zatsiorsky et al. (1979) in which they measured the start times, velocities of the centre of mass at different phases and forces applied during the block time.

Analyses show that the racing start technique in swimming remains very debatable among coaches, competitors and researchers. During the past few decades, different start modes have been investigated and compared (Issurin and Verbitsky, 2002). Some suggested that the grab start is superior to the track start (Ayalon et al., 1975; Zatsiorksy et al., 1979; Counsilman et al., 1988); others found no difference between the grab and track starts (Shin and Groppel, 1986; Kirner et al., 1989; Jorgić et al., 2010); and a third group of studies showed the track start to be superior to the grab start (Juergens, 1994; Allen, 1997; Holthe and McLean, 2001; Issurin and Verbitsky, 2002).

In three of the four racing styles of swimming — freestyle, breaststroke and butterfly — the start takes place above water, while the fourth, the backstroke, begins from inside the water. Another factor that affects the performance at start-up is the turn and the height of the starting block according to the FINA regulations (Pereira et al., 2002). According to Hay (1986), the start made out of the water is divided into three phases. The first phase includes actions by the swimmer on the start block once given the signal to start up until taking off of the start block. The second phase, called the flight phase, begins once the first phase is completed and ends when the first contact with water is made. The third phase, called the slip phase, begins the moment the second phase concludes and ends the moment the swimmer begins to swim normally. The success of the start-up depends on the successful execution of each phase separately, and the success of each phase depends on the proper execution of the previous phase. Therefore, most coaches and swimmers try to improve the first phase of start-up. The requirements for a superior start include a fast reaction time, significant jumping power, a high take-off velocity and a decrease in drag force during entry. A low resistance streamline position during underwater gliding to minimise the loss of horizontal velocity and an increase in propulsive efficiency during the transition stage can assist in a superior start (Schnabel and Kuchler, 1998; Breed and Young, 2003; Honda et al., 2010). The start of swimming is a jump forward to rapidly detach from the start block with the perfect angle and the maximum speed to cover a maximum distance. Apart from the take-off speed, there are other factors in the individual phases that may determine the outcome. The most important are the inclination of the orbit of the centre of mass to the horizontal level during take-off (angle off of the centre of mass), the spin that the body has during the take-off and the inclination of the orbit of the centre of mass from the horizontal level when contacting the water (entrance angle of the centre of mass) (Nikodelis and Kollias, 2003).

The aim of this study was to determine the differences in the kinematic parameters between the grab and track starts and the differences in these two starts between the genders.

## Material and Methods

### Participants

The sample consisted of 27 active swimmers at a competitive level, including 13 boys (mean ± SD: age 15.8 ± 0.8 years, body mass 67.7 ± 7.7 kg and body height 178.6 ± 5.7 cm) and 14 girls (mean ± SD: age 16 ± 0.8 years, body mass 59.2 ± 6.6 kg and body height 166.2 ± 6.7 cm).

First, all participants were informed by means of brochures, and parental consent was given for videotaping their children. All the participants’ parents provided written consent after being informed of the test protocol. The protocol of the study was approved by the Ethical Committee of the Faculty of Physical Education and Sports, Department of Aquatic Sports, National and Kapodistrian University of Athens and according to the revised Declaration of Helsinki.

The investigation was performed during the summer of 2011, when the swimmers were in the competitive period. During this period, all of the swimmers carried out the training program and participated in competitions at the international level. The coaches were carefully informed about the experimental procedures and the possible risk and benefits of the project.

The study protocol was held for every subject. In addition to the results, the basic anthropometric parameters (body height and body weight) and the age of the swimmers were registered in the study protocol.

### Measures

Flight distance (FD): The distance covered by the swimmer from the block until his hand enters the water, expressed in metres (m).

Flight time (FT): The time between leaving the block and the first contact of the swimmer’s hand with the water, expressed in seconds (s).

Flight velocity (FV): An indirect measurement calculated from the distance phase and flight phase with the equation FV=FD/FT and expressed in metres per second.

The entry angle (EA): The angle between the horizontal axis and the body. This angle was quantified at head entry (angle between the horizontal axis, the head and the hip), expressed in degrees.

Reaction time (RA): The time between the starting signal and the moment when the swimmer’s feet leave the block, expressed in seconds.

Body height and body mass were measured according to the instructions of the International Biological Program–IBP (Weiner and Lourie, 1969). Body height was measured with a GPM anthropometer (Siber and Hegner, Zurich, Switzerland) to the nearest 0.1 cm. Body mass was obtained by a precision scale (Bilance SALUS, Milan, Italy) to the nearest 0.1 kg.

### Procedures

#### Swim trial

During training, the swimmers were recorded in groups of two persons. Initially, the first was in one type of start, the grab start, and then relaxed during a 50 m swim. The second swimmer was recorded for the first type of start, and when he was resting, the first swimmer was recorded for the other type of start, the track start. Once he finished, we recorded the second swimmer. The same procedure was followed for the remaining swimmers. Each swimmer performed a standardised 15-minute warm-up consisting of a general easy swim before the testing. Three measurements were executed on the sample of 27 swimmers of freestyle and butterfly. Each swimmer made three starts for each type of start, for a total of six starts, and the best times for each start technique were analysed.

#### Video analysis

One lateral video camera (50 Hz, Panasonic NV-MS1 HQ S-VHS; Panasonic, Paris, France) was placed 5 metres from the edge of the pool and was used to videotape the block and flight phases. The videos were used to measure the entry angle, flight time, flight distance, flight velocity and reaction time.

The videotapes were digitalised with Human software (Human, version 6.0, HMA Technology Inc., 2005, Canada) at a frequency of 50 Hz. The reliability of the digitisation was assessed by digitising the 3 times for the 3 trials of all swimmers, and the average error of digitisation was 3.34%.

### Statistical Analysis

The Statistical Package for Social Studies SPSS (v17.0, SPSS Inc., Chicago, IL) was used for the statistical analysis. Descriptive statistics were calculated for all experimental data and reported as means ± SD. The Shapiro-Wilk test (p < 0.05) was used to test the normality of the distribution, whereas Levene’s test was used to test the homogeneity of the variance (Stone and O’Bryant, 1984). To determine the differences in the kinematic parameters between the two starting techniques and to determine the differences in both starting techniques between men and women, we used an ANOVA. The statistical significance was set at p < 0.05.

## Results

The Shapiro-Wilk test showed that the data were normally distributed. Levene’s test showed no violation of the homogeneity of variance. There is no statistically significant difference in the measured parameters between the grab and track starts between the males and females (Table 1). The males had greater numeric results for all of the kinematic parameters for the grab start compared with the track start, except for flight velocity and entry angle (flight time 0.42 vs. 0.41 s, flight distance 3.21 vs. 3.14 m, flight velocity 7.76 vs. 7.83 m/s, entry angle 44.22 vs. 43.85 degrees and reaction time 0.86 vs. 0.81 s). The females also had higher numeric results for all of the kinematic parameters for the grab start compared with the track start, except for flight time (flight time 0.38 vs. 0.38 s, flight distance 2.82 vs. 2.73 m, flight velocity 7.47 vs. 7.31 m/s, entry angle 45.18 vs. 44.79 degrees and reaction time 0.88 vs. 0.82 s).

The males had statistically better results for flight time and flight distance compared with the females for the grab start (FT 0.42 vs. 0.38 s, FD 3.21 vs. 2.82 m; Table 2). In the case of the track start, the males had statistically better results for flight distance (3.14 vs. 2.73 m).

## Discussion

The aim of this study was to determine the differences in the kinematic parameters between the grab and track starts and the differences between these two starts in terms of gender. The results of this research indicate that the males had a statistically longer flight distance for both start techniques compared with the females (p = 0.00). The FD was greater by 0.39 m for the grab start and 0.41 m for the track start. The males also had a significantly longer flight time for the grab start compared with the females (p = 0.04). The FT was longer by 0.04 s. No significant differences were determined for the remaining parameters. Furthermore, Nicholas and Watkins (2006) determined that the flight time recorded for the females in their sample was significantly shorter than the time recorded for the males. They tested 14 swimmers of both genders, whose ages ranged from 16 to 19 years. In addition to the FD and FT, the males also had a greater flight velocity (FV) compared with the females, but this difference was not statistically significant. In terms of the entry angle (EA), there was no statistically significant difference, and the entry angle was somewhat greater in the females compared with the males. In the research of Allen (1997), the females also had a greater entry angle compared with the men for both starting techniques, which was statistically significant. The females had greater numeric results for reaction time, which actually means that the males were better in this parameter because they left the starting block sooner.

In terms of the differences in the kinematic parameters regarding the gender of the swimmers, these differences can be based on the physiological differences that exist between the two genders. According to Beunen and Malina (2008), after the age of 14, the average physical performances of girls are consistently beyond the bounds of 1 SD below the means of boys in most tasks requiring speed, agility, balance, explosive strength, local muscular endurance, and static muscular strength, except flexibility. Male swimmers generally have a better swimming performance and achieve better results than female swimmers. This observation was also confirmed in the analysis of the results collected for the disciplines of 50, 100 and 200 metre freestyle in the Olympic Games in Barcelona, where the authors determined that the men possessed longer stroke lengths and started, turned, and swam faster than the women (Arellano et al., 1994). Male swimmers also have different physiological and metabolic parameters after the race and different technico-tactical characteristics than female swimmers (Mason and Cossor, 2000; Thanopoulos, 2010).

With respect to the measured kinematic parameters of the grab and track starts, the results obtained in this study indicate that there is no statistically significant difference between the two starting techniques. The males had greater numeric results for all of the kinematic parameters for the grab start compared with the track start, except for flight velocity and entry angle. The females also had greater numeric results for all of the kinematic parameters for the grab start compared with the track start, except for flight time. In the case of flight time, the obtained results match the results of previous research (Blanksby et al., 2002; Jorgic et al., 2010; Kruger et al., 2003; Miller et al., 2003). In a study involving 12 elite-level swimmers aged 17.7 ± 4.2, Blanksby et al. (2002) determined that there was no difference in the flight time between the grab and track starts. The aim of their study was to determine the influence of training on the improvement in the performance of the start technique. Both prior to and following the experimental program, the swimmers had greater means (mean) for FT in the grab start compared with the track start, but the difference was not statistically significant. The difference in the FT was 0.02 s prior to and 0.01 s following the experimental treatment. In the study carried out by Jorgic et al. (2010), the difference in the FT was 0.10 s between the two starting techniques but was also not statistically significant. In that study, the participants were Greek male swimmers with an average age of 15. Miller et al. (2003) also determined that there is no statistically significant advantage in flight time between the grab and track starts. The study was performed on 15 collegiate swimmers. Kruger et al. (2003) examined the differences in the two starts on a sample of women and concluded that flight time did not differ between the two starts.

The obtained results in terms of flight distance match the results of other studies (Takeda et al., 2006; Blanksby et al., 2001; Jorgic et al., 2010). In these three studies, there was no statistically significant difference in the flight distance between the two starting techniques. In a study based on a sample of 12 elite competitive swimmers, Takeda et al. (2006) determined that there was no statistically significant difference in the flight distance between the grab and track starts, with the flight distance being greater for the grab start than the track start (3.25 vs. 3.15 m). Jorgic et al. (2010) found a greater flight distance for the grab start compared with the track start (by 0.23 m) but no statistically significant difference.

Miller et al. (2003) determined a greater (p < 0.001) flight distance for the grab start compared with the track start, a difference that was statistically significant and measured 0.14 m. Vilas-Boas et al. (2002) also determined a greater flight distance for the grab start, which contrasted with the results given by Breed et al. (2000). Flight velocity was greater for the males in the track start compared with the grab start, while the situation was reversed for the females. Considering that flight velocity was calculated on the basis of the quotient between flight distance and flight time, the obtained results for the males and females were expected. In terms of the entry angle, no differences were determined between the two starting techniques. These results matched those from the research of Holthe and McLean (2001), who determined that very small differences could be found for the entry angle (EA) between the grab and track starts. On the basis of this finding, the authors concluded that the swimmers have the ability to practice and perform the appropriate start into the water under the appropriate angle for both start techniques. Vilas-Boas et al. (2002) also determined that there was no difference in the entry angle between the two start techniques. Unlike these studies, Miller et al. (2003) determined a significant difference in the entry angle between the grab and track starts in which the entry angle was smaller for the grab start.

In both males and females, the reaction time was greater for the grab start compared with the track start. In the case of reaction time, the aim of the swimmers was to leave the starting block in as short a time span as possible. Thus, lower numeric values for reaction time for the track start actually represent better results compared with those achieved for the grab start, i.e., the swimmers were able to leave the starting block much sooner. Allen (1997) also determined that the participants left the starting block sooner for the track start compared with the grab start, which is usually ascribed to the lower position of the COG of the swimmer on the starting block. Issurin and Verbitsky (2002), while analysing the differences between the grab and track starts at the Olympic Games in Sydney, determined a significantly quicker take-off from the starting block in almost all the events. Blanksby et al. (2002) did not determine any differences in the speed of the take-off from the starting block between the two starting techniques.

The starting technique is always born from the attempt to determine the differences between successful and unsuccessful attempts, with the style of the individual technique obtained from the successful attempts. Because this research showed that there were no significant differences for the analysed parameters (FT, FD, FV and EA), which represent the flight phase and entry phase, analysing the take-off from the starting block is necessary because the flight and entry phases are only the consequence of contact with the surface (the take-off phase from the starting block). By studying reaction time as one of the parameters of the take-off phase, we can determine that in the case of these swimmers, the track start provides a quicker take-off. According to Issurin and Verbitsky (2002), reaction time is significantly correlated with start efficiency, i.e., the duration of the swimming up to 15 m following the start. Thus, more work needs to be done to improve RT. Although numerically speaking, the track start allows a swimmer to leave the starting block sooner, but the obtained differences were not significant. In accordance with these findings in the group of male and female swimmers studied herein, we cannot speak in favour of the track start.

For any future research, analysing the phase of transition into swimming (underwater phase following entry) is also important, which may represent the most important phase that connects the start (the beginning of the race) with the remaining elements of the race (distance swimming, the swimming phase prior to the turn, the turn itself, the transition into swimming, the distance swimming and the finish) in the example of the 100 m race in the long swimming pull. According to Sanders and Bonnar (2008), there is evidence that the underwater phase that follows entry is of great importance for the analysis of start techniques. Exploring the characteristics of the two starts did not lead to any significant kinematic differences. Therefore, a conclusion that demonstrates the superiority of one of the start techniques cannot be reached. This finding is in agreement with a great number of studies that also did not confirm an advantage of one start technique over another. The coach, together with each swimmer individually, should devote some time to decide after some tests what type of start best fits the body type and general qualifications of the swimmer. When choosing a starting technique, certain rules should be adhered to. According to Lyttle and Benjanuvatra (2004), a swimmer who has a large asymmetry in terms of force production (one leg produces significantly more force than the other) should practice and use the track start with the dominant force-producing leg forward. In contrast, swimmers who are very explosive and symmetrical in their force production should practice and perform the grab start in which they can produce very high force levels in a short period of time. Afterwards, the swimmer should specialise in the chosen start and try to improve the weakest points in his technique.

## Figures and Tables

**Table 1:** Differences between the grab and track starts for male and female swimmers (mean ± SD)

	**Males (*n*=13)**		***p***	**Females (*n*=14)**		***p***
	**Grab start vs. Track start**			**Grab start vs. Track start**		
Flight time (s)	0.42 ± 0.06	0.41 ± 0.07	0.63	0.38 ± 0.05	0.38 ± 0.06	0.97
Flight distance (m)	3.21 ± 0.25	3.14 ± 0.20	0.43	2.82 ± 0.21	2.73 ± 0.21	0.25
Flight velocity (m/s)	7.76 ± 1.10	7.83 ± 1.19	0.88	7.47 ± 0.69	7.31 ±0.99	0.61
Entry angle (degree)	44.22 ± 5.58	43.85 ± 4.48	0.85	45.18 ± 4.02	44.79 ± 4.00	0.80
Reaction time (s)	0.86 ± 0.09	0.81 ± 0.07	0.14	0.88 ± 0.13	0.82 ± 0.08	0.17

* statistically significant, p<0.05

**Table 2:** Differences between male and female swimmers for both start techniques (mean ± SD)

	**Grab start**		***p***	**Track start**		***p***
	**Male vs. Female**			**Male vs. Female**		
Flight time (s)	0.42 ± 0.06^*^	0.38 ± 0.05^*^	0.04	0.41 ± 0.07	0.38 ± 0.06	0.28
Flight distance (m)	3.21 ± 0.25^*^	2.82 ± 0.21^*^	0.00	3.14 ± 0.20^*^	2.73 ± 0.21^*^	0.00
Flight velocity (m/s)	7.76 ± 1.10	7.47 ± 0.69	0.42	7.83 ± 1.19	7.31 ± 0.99	0.23
Entry angle (degree)	44.22 ± 5.58	45.18 ± 4.02	0.61	43.85 ± 4.48	44.79 ± 4.00	0.57
Reaction time (s)	0.86 ± 0.09	0.88 ± 0.12	0.72	0.81 ± 0.07	0.82 ± 0.08	0.79

* statistically significant, p<0.05
